# Frontline clinical diagnosis—FTIR on pancreatic cancer

**DOI:** 10.1002/cam4.6346

**Published:** 2023-07-19

**Authors:** Edward Duckworth, Matthew Mortimer, Bilal Al‐Sarireh, Venkateswarlu Kanamarlapudi, Debdulal Roy

**Affiliations:** ^1^ Swansea University Swansea Wales UK; ^2^ Morriston Hospital Morriston Wales UK

**Keywords:** biomarker, cancer, diagnosis, FTIR, pancreatic, PCA, spectroscopy, SVM

## Abstract

**Objective:**

Accurate, easily accessible and economically viable cancer diagnostic tools are pivotal in improving the abysmal 5% survival rate of pancreatic cancer.

**Methods:**

A novel, affordable, non‐invasive diagnostic method has been developed by combining measurement precision of infrared spectroscopy with classification using machine learning tools.

**Results:**

Diagnosis accuracy as high as 90% has been achieved. The study investigated urine and blood from pancreas cancer patients and healthy volunteers, and significantly improved accuracy by focusing on sweet‐spots within blood plasma fractions containing molecules within a narrow range of molecular weights.

## Graphical abstract

The graphical table of contents depicts the flow of cancer information, data acquisition and diagnosis.

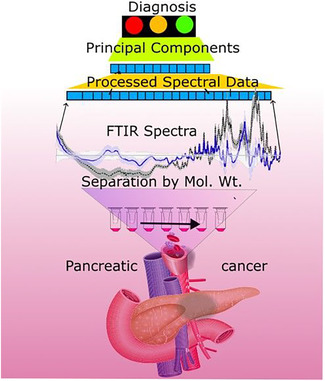

## 1 INTRODUCTION

Pancreatic cancer is one of the most deadly cancers in the UK,^1^ the 5‐years survival rate remaining below 10%.^2^ Nearly half of people born in England since 1960 will receive a cancer diagnosis in their lifetime.^3^ This is mostly due to that it is hard to detect early. This cancer is very suitable for investigation as this type of hidden cancer requires the most diagnostic aid, as it is unlikely to be easily diagnosed by any other means as notable symptoms (e.g. abdominal pain and jaundice) typically only occur once the cancer has reached a late stage.^2^ The best way to improve the survival rate, and also thereby reduce the treatment costs,^3^ is to have a viable method of effectively screening for the condition early in its onset. Screening has been proven to be effective on other cancers in the UK.^4^ An effective screening method needs to be fast, affordable, non‐invasive and simple enough to not require a clinician at all stages.^5^ This raises the question: is there a bio‐fluid spectroscopy technique that can provide the solution? By comparing a scan of an unknown sample with an organised database of known healthy and cancerous samples, spectral biomarkers can be used to determine whether there is an affliction.^5^, ^6^, ^7^

Blood plasma is a particularly useful bio‐fluid for inspection due to its high protein and lipid concentration.^6^, ^8^

**FIGURE 1:**
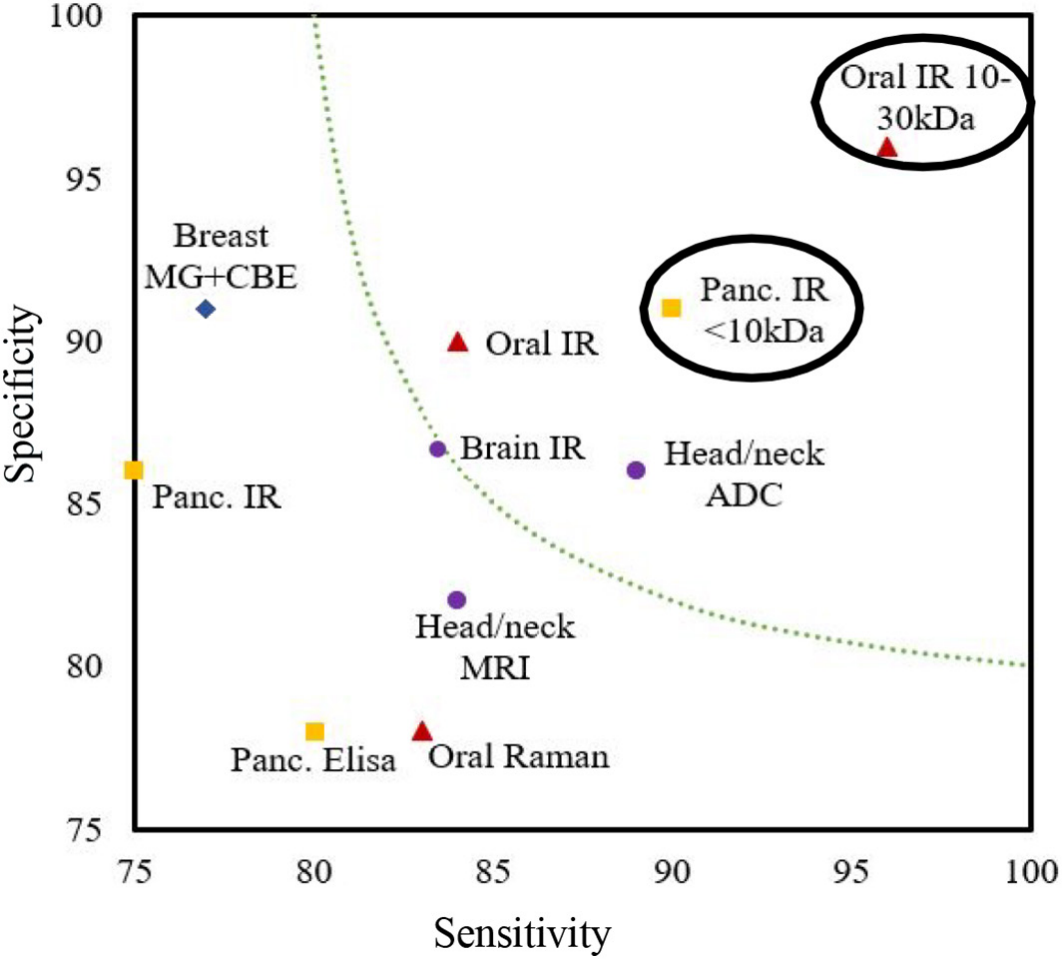
Summary of cancer diagnostic techniques. High lighted by circles, Pancreatic cancer work (shown as ■) and oral cancer work (shown as ▲) on plasma are from our research group on 74 pancreatic and 120 oral cancer patients.^15^ Raman data are also from our own research on 90 patients' serum, though the 10–30 kDa variation is on an 18‐patient subset. Head/Neck/Brain cancer data from a recent study by Van Der Hoorn et al.^20^ using 854 patients for the MRI and 287 for the apparent diffusion coefficient (ADC) imaging metric and clinical validation result from an IR brain cancer study by Butler et al.^10^ (shown as ●). Mammogram (MG) combined with clinical examination (CBE) were from a breast cancer (shown as ◆) study on 32,080 patients by Noriaki et al.^21^ The dotted curve represents a qualitative boundary of acceptability followed by us with a minimum 85% accuracy. Accuracy is defined as the % of cases classified correctly.

Vibrational spectroscopy as a potential method to diagnose cancerous patients has been frequently explored over the past decades, utilising many varied methodologies.^6^, ^8^, ^9^, ^10^ Figure 1 compares the laboratory‐based vibrational spectroscopy methods with commonly used mammogram and magnetic resonance imaging (MRI). There is excellent potential in this field of research to produce an accurate, non‐invasive detection method when used to analyse key human biofluids like blood, saliva or urine.

Reviews on the recent literature highlighted that promising research had already been done on pancreatic cancer, concluding that FTIR was of most use of clinical applications due to its speed. Raman was deemed useful to allow deeper investigation into subcellular mechanisms.^2^, ^11^ However, it was also concluded that more research was still necessary with larger sample cohorts and better ability to handle irregular cases and exceptions.

Amongst the vibrational spectroscopy instruments, the Fourier Transform Infrared (FTIR) spectroscopy offers an economically viable opportunity due to its low set up cost.^2^ If measurement costs could be minimised, this would then allow for more readily available screening for these diseases, leading to earlier detection overall.

Many of the investigations only demonstrate the potential of a particular method to produce a spectral biomarker between diseased and healthy samples on a relatively small sample set. In one exemplar study, FTIR was used on human blood serum and plasma looking at colorectal cancer.^12^ The study identified deviations in certain peaks in plasma and serum between healthy and cancerous samples. Due to presence of a large variety of molecules, it is extremely difficult to assign a specific peak to the responsible biomarkers. With the advancement of machine learning tools, it is now possible to analyse the spectra with significantly higher accuracy. One study deployed statistical methods to analyse FTIR spectra of non‐small cell lung carcinoma serum to distinguish between patients with cancerous and non‐cancerous lung diseases and healthy volunteers^13^ and achieved 80% accuracy.^14^

Another study on brain cancer^10^ initially achieved 93/92% sensitivity specificity, though this reduced to 83/87% when validating clinically. This is still a valuable diagnostic test, but much reduced from the testing performance. The key difference between the testing and validation cohort was the non‐cancer control. When initially selecting, they used serum from healthy patients with no‐cancer symptoms or background, whereas when validating they were testing against people called in for brain imaging for a suspected cancer.

A recent study by our research group^15^ identified a novel method for detecting cancer patients in an oral cancer based study. It achieved good success from filtering the patients' blood into molecular weight sections before examining each with FTIR, reducing the sample's complexity and removing obscuring signals from molecules like globulin (>80 kDa) and albumin (>60 kDa).^16^ Henceforth, a *molecular‐weight window* would refer to the set of molecules whose molecular weights lie between an upper and lower cut‐off limits. This novel methodology identifies a *sweet‐spot* or the molecular‐weight window with high classification accuracy for a group of patients' diseases and discards molecules outside the window. Focusing on a narrow molecular‐weight window provides the precision and enables detection of the spectral changes due to a specific disease.

The goals of this research are as follows:

1. To improve accuracy and reduce variability in the diagnosis of pancreatic cancer using blood and an affordable spectroscopy method.
2. To investigate the suitability of noninvasively obtainable bio‐fluid, urine, for spectral diagnosis using this method.

## 2 EXPERIMENTAL METHOD

### 2.1 Study design and sample preparation

(Figure S1). Instead of a large cohort of patients, which requires significant resources, this study was designed on the principle of achieving ‘statistical precision’ from ‘measurement precision’ from a small cohort size to develop the underpinning method.

Blood plasma and urine samples were collected with ethical approval from the same cohort of patients from Morriston hospital, Swansea, UK between 2020 and 2022. IRAS ID: 252525. A full list of patients is given in Supplementary Information (Table S1). For the full cohort plasma experiment, 17 had late‐stage pancreatic cancer (C), 14 had early‐stage (EC), 10 were healthy (H) and 31 had premalignant pancreatic conditions (P). For the molecular‐weight windowing experiment, nine patients had advanced pancreatic cancer and were compared to nine other patients who had premalignant pancreatic conditions. Samples were stored frozen until being thawed for analysis emulating transporting samples from hospitals and triages to elsewhere for analysis. This work aimed at establishing the accuracy of FTIR diagnosis in comparison with CA19‐9 tests using ELISA.

For the FTIR measurement, each fraction was diluted in a 1:24 ratio of sample to MilliQ ultrapure water before 500 μL being deposited on a 25 mm diameter Crystran CaF2 slide, ensuring the surface was covered to the edges and left to dry overnight for analysis. Each biofluid sample was first filtered through a 100 kDa filter, both filtrate (permeate) and concentrate (retentate) being collected, and the filtrate being moved on to further filtering using 50, 30, 10 and 3 kDa filters until six subsets of the plasma samples were produced. The subset windows 0–3, 3–10, 10–30, 30–50, 50–100, >100 kDa and whole plasma were all analysed for comparison. For the full cohort, only a 10 kDa filter was used, both whole and< 10 kDa plasma being analysed.

### 2.2 Spectral acquisition

FTIR spectra were acquired with a Perkin Elmer ‘Spectrum Two’ FTIR spectrometer used in transmission mode. Resolution was 4 cm^−1^, and spectra were acquired for 5 s with 10 accumulations over a range of 750–4000 cm^−1^.

### 2.3 Pre‐processing

Spectra were trimmed to the 1000 data points in the 800–1800 cm^−1^ fingerprint region of most interest and then pre‐processed with a background correction using the asymmetric least squares smoothing (ALSS) method.^17^ This was followed by average normalisation.

### 2.4 Model development

We collected patient samples with 1000 dimensions (one intensity value per wavenumber in the 800–1800 cm^−1^ range) and classified these samples with Support Vector Machine (SVM) classifiers. We used a linear SVM classifier with and without PCA. As we have more features than samples, which can sometimes lead to overfitting, we used PCA to reduce the number of dimensions in the original data. The results were validated with complete Leave‐One‐Out (LOO) cross‐validation. As the combination of PCA and SVM provided the best results, we also studied the cross‐validated accuracy of the classifier according to the number of principal components used. Representative spectra are given in S‐7 for comparison. Classification accuracy was also obtained from spectra for each of the different molecular weight fractions (Figure S2 and S3). The classification model is compliant with the Data Optimisation Model Evaluation (DOME) standard.^18^ Confidence values for each classification were produced using 95% Clopper–Pearson confidence intervals.^19^

## 3 RESULTS

3.1

Classifying cancer and healthy patients is relatively straight forward. We believe that the main challenge lies in eliminating the standard inflammatory and other general disease markers from the premalignant conditions that deteriorate the diagnosis accuracy of cancer as both cancer and premalignant patients reach out to clinicians with similar symptoms. Therefore, we focused on classifying the hardest to discern, that is cancer and premalignant conditions. For screening and potential quantification purposes in the full cohort study, a healthy control set was also used.

**FIGURE 2:**
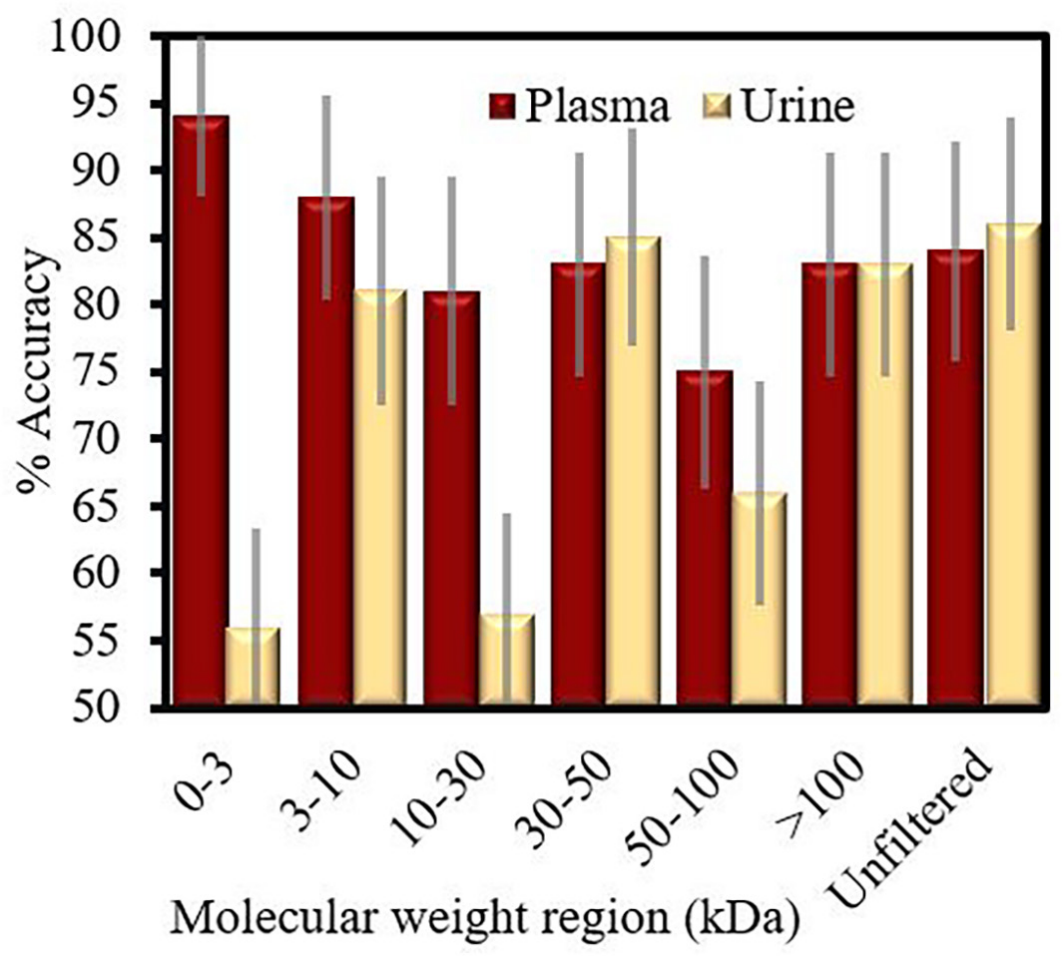
Classification accuracies between FTIR spectra of pancreatic premalignant and cancer patients for different plasma and urine molecular weight subsets. Errors are standard binomial error calculations.

In the blood plasma *molecular‐weight windowing* study, as shown in Figure 2, the lower molecular weight regions produced the highest cross‐validated classification accuracies. The most accurate was the <3 kDa region, with 94% accuracy, followed by the 3–10 kDa at 88%. Both were higher than the classification for whole pancreatic cancer plasma, which scored 84%. The urine data (Figure 2) performed relatively worse overall. Unfiltered urine, >100 kDa filtrate and 30–50 kDa filtrate scored around of 86%. The cohort size for this preliminary experiment was quite small, resulting in the larger 95% confidence of 5% for the most effective groupings, up to 10% for those with less accuracy. These can be seen on Figure 2.

As the two highest scoring regions were the <3 kDa and the 3–10 kDa, for practical purpose and ease of implementation, we designed our study to probe the <10 kDa plasma filtrate, with a comparison to whole plasma. From Table 1, we can see that this <10 kDa filtrate performed better than whole plasma for most comparisons made, each has 90% or higher accuracy when diagnosing late‐stage patients against healthy and premalignant pancreatic patients (See Table S2 for an example confusion matrix of this classification). Furthermore, when early‐stage cancer patients were included, 90% accuracy was still achieved. The ability to distinguish between early and late‐stage cancer patients with a 90% accuracy is also demonstrated.

**TABLE 1:** Comparison of plasma diagnostic accuracies after PCA‐SVM and LOO‐cross‐validation on the subsets in the study.

Fraction	Subsets compared	Sens. (%)	Spec. (%)	Acc. before CV (%)	PCA‐SVM Acc. (%)	95% Confidence interval (%)	SVM only Acc. (%)
Whole plasma	C v P	74	90	91.6	**84.3**	77.3–90.4	75.3
	C v H	100	100	100	**100.0**	95.6–100.0	92.4
	C + EC v H + P	75	86	100	**81.3**	75.4–86.2	75.7
	C v EC	88	78	100	**83.5**	74.4–90.4	76.9
<10 kDa window	C v P	90	90	100	**90.0**	84.5–95.1	86.5
	C v H	97	93	100	**95.3**	87.8–99.0	87.2
	C + EC v H + P	90	91	100	**90.6**	85.7–94.3	89.2
	C v EC	90	90	100	**90.0**	80.6–95.8	58.6

*Note*: Cross‐validated SVM accuracy included for comparison. Table S3 contains additional information. This is the main accuracy of interest. It is cross validated and is an appropriately weighted combination of sensivity and specificity (in bold).

Abbreviations: C, Cancer; EC, Early‐stage Cancer; H, Healthy; P, Premalignant.

Once can also see that by using PCA‐SVM, over SVM alone, the accuracy after cross‐validation is improved due to there being less overfitting.

**FIGURE 3:**
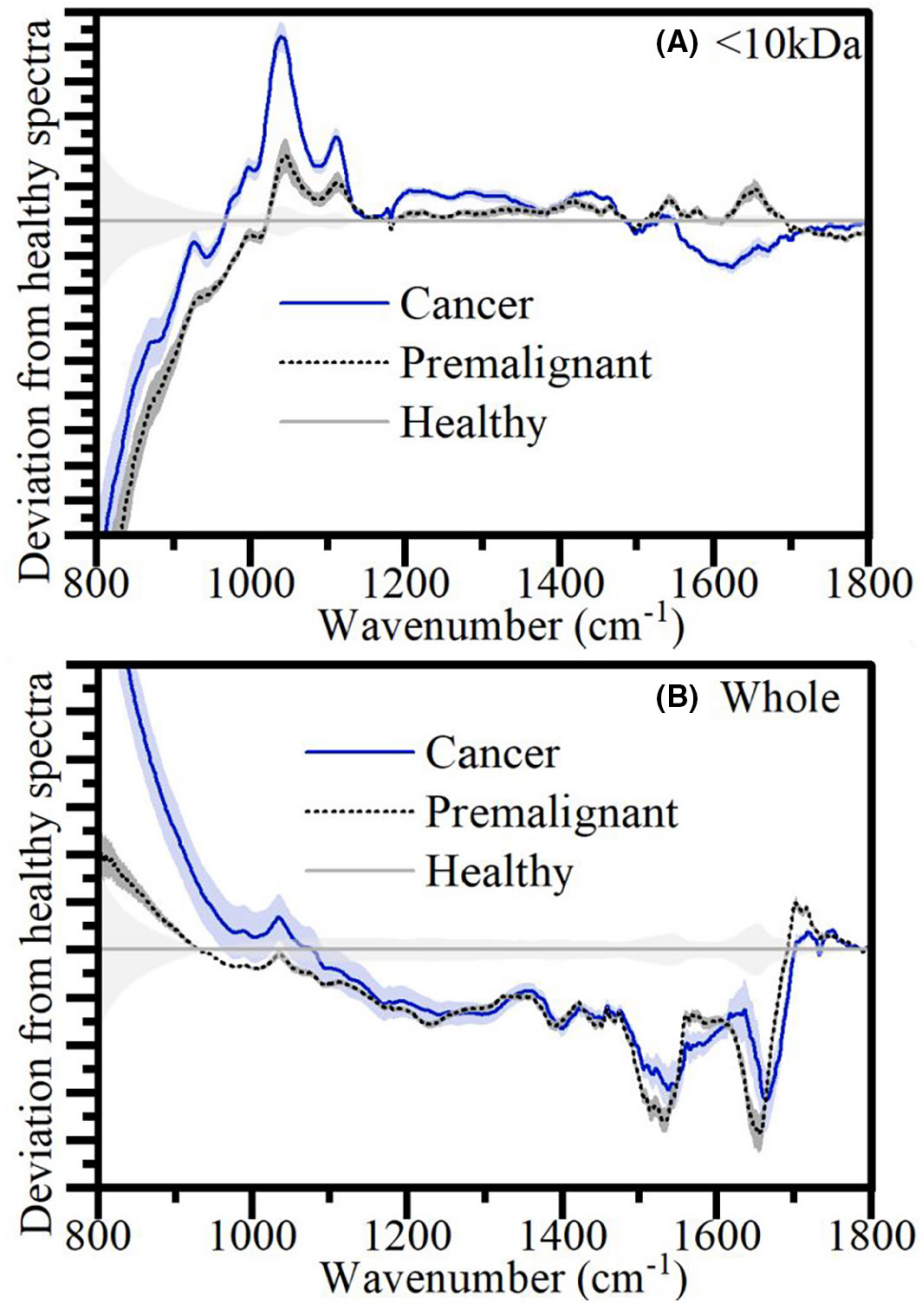
Difference in the average spectra of cancer and premalignant patient serum from healthy. (A) <10 kDa, (B) whole serum. Faded colour around each line shows the spread for different patients.

In Figure 3, one can see the average spectral differences between the diagnosis categories for the <10 kDa and the whole plasma. As it is hard to distinguish the full average spectra by eye, the difference from the healthy spectra is shown highlighting the key peak shifts in unhealthy patients. It is important to note that the differences discerned by the eye‐test in these average spectra, though they may make up part of the classification, it is the less obvious shifts that are picked up by the PCA‐SVM analysis that contribute to the higher % accuracy. Regardless, we can get some indication of the biomarker's origins from these visual observations. One can see how in the whole set, the difference between premalignant and cancer is less obvious than in the <10 kDa, which is reflected by the higher accuracies in Table 1. This contributes to the theory that removing the higher weight components reduces confounding information and highlights the usefulness of this key molecular weight region of interest. From this <10 kDa region, of note are the peaks shifts at 1050 cm^−1^ and 1600 cm^−1^. See Figure S3 for the molecular windowing experiment spectra.

## 4 DISCUSSION

Distinct improvement in accuracy can be clearly observed for the <10 kDa plasma window in distinguishing cancer. The classification of the FTIR spectra was 6%–9% higher than whole plasma for most diagnostic comparisons, achieving 90.6% accuracy for cancer v all non‐cancer controls.

It is worth noting that whole plasma performed better than <10 kDa to distinguish healthy class. This is the least important classification to achieve, as it is likely to be affected by noncancer factors. Furthermore, the values for classifying Cancer v Healthy were high in both weight classes, and within confidence of one another.

Unlike in the study by Butler et al,^10^ we reinforced an effective control, especially a ‘related disease’ control, as ‘premalignant’ in our study. One can see the reduction effectiveness in this study when classifying between cancer v healthy (95.3%) and cancer v premalignant (90%).

This study's results can be compared to currently used ELISA methods using the known pancreatic cancer biomarker Carbohydrate Antigen 19–9 (CA19‐9, molecular weight 820 Da), which produced 70%–80% accuracy on the same patient samples (Figure 1). This biomarker is in the <10 kDa region used. However, the patients misclassified by each method were different. Attempts were made to establish if ELISA and the FTIR method are probing the same biomarkers by measuring the <10 kDa filtrate using both methods. In our study, ELISA consistently reported lower level of CA19‐9 in the <10 kDa filtrate than the whole plasma. This clearly indicates that the spectral biomarker is unrelated to CA19‐9, and CA 19–9 is either not contributing significantly to the IR signal, or it is attached as glycoprotein and is filtered out with high molecular weight molecules.

The key strength exhibited in this study is the use of suitable controls and a robust methodology. This methodological precision resulted in high classification accuracies between difficult to discern diagnoses, indicating that the test could be an extremely valuable addition to clinical practice. However, it is still limited by cohort size and the use of only one testing site. It is required to expand the study to a larger cohort over multiple sites to achieve clinical validation.

## 5 CONCLUSION

From these results, one can conclude that using the <10 kDa molecular weight region can provide a practical and superior classifier model to using unfiltered plasma alone. Furthermore, urine could be used as a diagnostic biofluid, but the accuracy would not be as high as when plasma is used. The additional ability to diagnose early‐stage patients only increases the potential for the method to be used for screening cancers before they can progress to late‐stage cancer. Furthermore, being able to discern early from late‐stage cancers with a high accuracy is also invaluable information for a patient's treatment.

We also deployed the molecular weight windowing method for diagnosis of oral cancer and observed significant improvement in diagnosis accuracy.^15^ One can see a summary of the pancreatic and these accuracies, along with the ones from practice and other promising research, in Figure 1. From these combined results, it is plausible that narrowing down the molecular weight window can allow us to probe the sweet spots to diagnose different types of cancer with high accuracy at an early stage.

## AUTHOR CONTRIBUTIONS

**Edward Duckworth:** Investigation (equal); software (lead); writing – original draft (equal). **Matthew Mortimer:** Formal analysis (equal); investigation (equal); methodology (equal); validation (equal). **Bilal Al‐Sarireh:** Conceptualization (equal); project administration (equal); resources (equal); writing – review and editing (equal). **Venkateswarlu Kanamarlapudi:** Formal analysis (equal); investigation (equal); methodology (equal); writing – review and editing (equal). **Debdulal Roy:** Conceptualization (equal); data curation (equal); formal analysis (equal); funding acquisition (equal); methodology (equal); project administration (equal); resources (equal); supervision (equal); writing – review and editing (equal).

## CONFLICT OF INTEREST STATEMENT

There are no conflicts to declare.

## ETHICAL APPROVAL AND CONSENT

Ethical approval was obtained from The Wales Research Ethics Committee (REC) 7 and the SBU Joint Scientific Review Committee. Written informed consent was obtained from the participants.

## Supporting information


Data S1.

## Data Availability

Not Applicable.
